# The Regulation of Micro-Organisms’ Extra-Cellular Polysaccharides on Immunity: A Meta-Analysis

**DOI:** 10.3390/foods11131949

**Published:** 2022-06-30

**Authors:** Jin Zhang, Yirui Chen, Jiaqi Zhang, Yitong Wang, Yanan Liu

**Affiliations:** 1 Department of Food Science and Engineering, Ningbo University, Ningbo 315211, China; zhangjin90317@163.com (J.Z.); zhang17815843173@163.com (J.Z.); wangyitong20010222@163.com (Y.W.); 2 Department of Genetics, Bioinformatics and Computational Biology, Virginia Polytechnic Institute and State University, Blacksburg, VA 24060, USA; yiruic@vt.edu

**Keywords:** extra-cellular polysaccharides, immunity, meta-analysis

## Abstract

Extra-cellular polysaccharides (EPSs) have excellent immunomodulatory functions. In order to further promote their application, we studied the ability of extra-cellular polysaccharides from different sources to regulate immunity. We studied the association of extra-cellular polysaccharides with immune factors (Interleukin (IL-2, IL-4, IL-10), Interferon γ (IFN-γ), tumor necrosis factor-α (TNF-α), Immunoglobulin A (IgA), and Immunoglobulin G (IgG)) and different concentrations of EPSs and interfering media on experimental results by using a forest plot under fixed-effect or random-effects models. Through Google, PubMed, Embase, ScienceDirect, and Medline, from 2000 to 2021, 12 articles were included. We found that exopolysaccharides (from bacteria or fungi) could significantly increase the immune index of spleen and thymus, spleen index (SMD: 2.11, ‘95%CI: [1.15, 3.08]’; *p* < 0.01), and thymus index (SMD: 1.62, ‘95%CI: [0.93, 2.32]’; *p* = 0.01 < 0.05). In addition, exopolysaccharides had a significant effect on TNF-α (SMD: 0.94, ‘95%CI: [0.29, 1.59]’; *p* = 0.01 < 0.05). For IL-4 (SMD: 0.49, ‘95%CI: [0.01, 0.98]’; *p* = 0.046 < 0.05), extra-cellular polysaccharides had a statistically significant effect on immunity. Although the data of other immune factors were not ideal, the comprehensive analysis showed that exopolysaccharides also had an effect on the release of these five immune factors. In the sub-group analysis, different concentrations of EPSs affected the results of experiments on the spleen and thymus, and the CY intervention had a relatively significant effect on immune regulation. Taken together, our study highlighted that EPSs have a significant impact on immune regulation.

## 1. Introduction

Extra-cellular polysaccharides (EPSs) are widely used in food because of their favorable shelf-life improvement properties, ability to improve food quality, and beneficial health effects. EPSs, as starter production agents, improve the water retention and overall rheological properties of yogurt [1,2]. EPSs, which can improve the quality of food, are thickeners, emulsion stabilizers, and some fat substitutes [3]. In addition, the use of extra-cellular polysaccharides to deliver anti-cancer drugs to target cells is also a hot topic in recent years, which has been warmly welcomed by the medical community [4]. EPSs act as immunomodulators, enhancing host immunity and killing invading bacteria, fungi, viruses, etc. [5,6]. Studies have shown that EPSs enhance immune tolerance to allergens and have long-lasting effects [7]. Probiotic polysaccharide capsules have anti-allergic effects, and polysaccharide capsules of *Lactobacillus* and *Bifidobacterium* can reduce serum levels of allergen-specific antibodies to slow down allergic reactions [8,9]. Some EPSs can be used as biosorbents and flocculants to decolor wastewater and remove heavy metals from industrial water to protect the environment [10]. Some microbial EPSs become potential templates for the rapid synthesis of metal nanoparticles, and the mediating process is simple and environmentally friendly [11]. Extra-cellular polysaccharides have anti-tumor effects, according to Tahmourespour et al. [12], the EPSs of Pseudomonas aeruginosa have a significant inhibitory effect on the HT-29 colon cancer cell line [13]. Additionally, extra-cellular polysaccharides also have some health functions, such as antioxidants, anti-cholesterol activity, and the characteristics of lowering blood pressure [14,15,16].

In various fields such as food, medicine, and others, extra-cellular polysaccharides have broad application prospects. However, there have been numerous studies reporting on the immunomodulatory functions of polysaccharides of different sources (molecular weight, monosaccharide composition, structural conformation, functional groups, etc.) [17,18]. Di et al. revealed that a 1–4 glycosides bond has anti-tumor effects [19]. Surayot et al. [20] found that low-weight–average-molecular-weight EPSs were more effective in stimulating macrophages than native EPSs. Some studies have shown that the related physiological activities of EPSs after chemical modification, such as a sulfate group, carboxymethyl group, phosphate group and acetyl group, can be greatly improved [5,21]. 

Extra-cellular polysaccharide immune function is of great value for the development of novel health foods. This paper ignores the influence of extra-cellular polysaccharide structure to analyze the overall immunomodulatory effect of extra-cellular polysaccharides. Here, we systematically integrated and analyzed 12 independent studies on the association between extra-cellular polysaccharides and immune regulators in a statistic manner. We hoped to verify that extra-cellular polysaccharides of different origins have certain immune efficacy, thereby lowering the application threshold of extra-cellular polysaccharides, to promote the further application of extra-cellular polysaccharides in the field of food. Some application areas include developing food with health care functions, continuously improving the level of human health in the diet, reducing the rate of illness, and promoting in-depth research on EPSs. 

## 2. Methods

### 2.1. Search Strategy

We conducted literature searches, systematic reviews, and reports according to the Preferred Reporting Project (PRISMA) scheme for systematic evaluations and meta-analyses [22]. We selected relevant keywords to search the Google, PubMed, Embase, ScienceDirect, and Medline databases: (immunity OR immune) AND (Exopolysaccharides OR EPS OR polysaccharides OR polysaccharose) AND (randomized OR randomized OR RCT). We included publications in English from 2000 to the 23rd of September 2021. 

### 2.2. Inclusion and Exclusion Criteria 

The inclusion criteria for the review were as follows: Studies were included based on pre-specified subjects, interventions, controls, outcomes, and study design (PICOS) criteria (Table 1). Review articles, conferences, abstracts, and articles that did not meet the criteria were not included. Data on inflammatory markers (immune indicators) as outcome measures were included in the analysis. Articles with immune indicators but incomplete data that still did not have complete information after contacting authors were excluded.

### 2.3. Selection Process

The title and abstract of the publication were screened in duplicate by two of the study’s workers (J.Z. and J.-Q.Z.), and the selected literature was summarized. Screening was then based on eligibility criteria, and studies were selected for extra-cellular polysaccharides applied alone and without treatment in control or placebo groups. Searches of the full text of all publications complied with the screening procedure, and papers were evaluated by two identical authors for a second screening. We excluded articles that was from the same author, had imperfect data, or had unitless measures in this screening, and any discrepancies in evaluation or decision making were resolved in discussions with another independent study investigator (Y.W.).

For the included articles, we chose to collect the following data: study identification (author, year), experimental subjects (category, sample size), intervention (type, duration of intervention, dose), control group, experimental design, results, and number of and variance of immune indicators before and after extra-cellular polysaccharide administration.

### 2.4. Quality Assessment

Two researchers assessed the quality of the included articles based on appropriate modifications of the STAIR list (J.Z. and J.-Q.Z.). The tool includes 9 entries including (1) calculation of sample size, (2) concealed experimental animal grouping scheme, (3) random sequence generation, (4) report the reason why animals were excluded from the analysis, (5) blinded evaluation of outcomes, (6) integrity of information, (7) declare potential conflicts of interest and research funding, (8) instructions for temperature control, (9) animal models (mice with drug-induced body damage). This article used a scoring system, and if the experiment published in the literature was carried out according to the content of the above entry, one point was added; otherwise, it received 0 points. If the final score was 1–3 points, the literature quality was poor; the articles with 4–6 points was medium; the articles with 7–9 points was the highest. If there were any disagreements about the evaluation indicators, a third party (Y.W.) was consulted.

### 2.5. Data Organization and Analysis

When calculating the overall effect size, we used a forest plot to visualize the aggregate results of the meta-analysis. Standardized mean difference (SMD) was used to estimate the effect size between groups. Hedges’ g and restricted maximum-likelihood estimator were used to measure pooled effect in small-sample-sized data and cross-studied variance, respectively [23]. To investigate the heterogeneity between studies, Q-test [24], I^2^ statistic [25], and H statistics [25] were implemented. For Q-test, if *p*-value is less than 0.05, there is heterogeneity; otherwise, it is considered to be non-heterogeneous. I^2^ > 50% means that there is statistical heterogeneity; otherwise, there is no heterogeneity. The value of H = 1 represents complete homogeneity; H > 1.5 indicates heterogeneity in the study. When H is between 1.2 and 1.5, and when the 95% confidence interval for H contains 1, it is impossible to determine whether there is heterogeneity at the test level of α = 0.05, and if it does not contain 1, heterogeneity can be considered. If there is heterogeneity among studies, a random-effects model is used; otherwise, a fixed-effects model is used. Further sub-group analysis was conducted on the effects of EPS concentration and experimental interference on immune results, and the significance test took two-sided *p* < 0.05 as the difference being statistically significant. A forest plot was used to visually display the statistical summary results of the meta-analysis.

## 3. Results

### 3.1. Basic Characteristics of Literature Search and Included Studies

**Figure 1:**
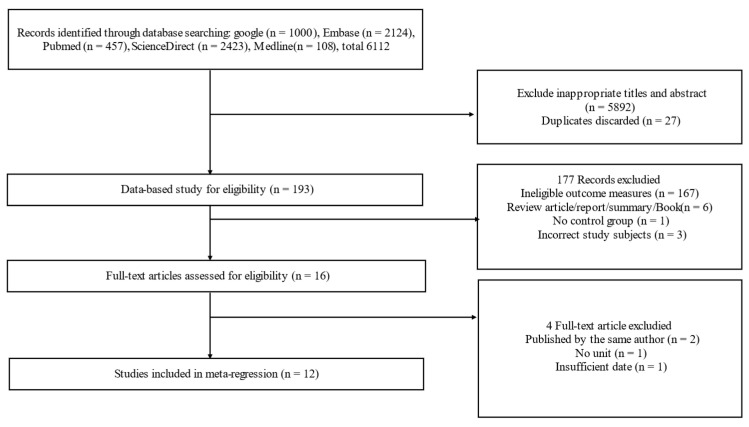
Preferred Reporting Items for Systematic Reviews and Meta-analyses (PRISMA) flow diagram of study selection.

As shown in Figure 1, we searched a total of 6112 references from the five databases: Google, PubMed, Embase, ScienceDirect, and Medline. By selecting using the title and abstract and excluding irrelevant articles, we obtained 193 relevant articles. According to Table 1, we excluded 177 references, of which 167 references had ineligible outcome measures. Six publications that did not meet the document type requirements were also removed. Articles without a control group were also not included. In addition, three articles with different research objects were removed, in which studies were on shrimp, sheep, and chicken, rather than mice and humans. In addition, EPSs coming from bacteria and fungi, rather than the sugars that some plants obtain by themselves, were not the focus of our study, and articles involving research on them were excluded as well. Finally, we removed one article with no units and one article with insufficient data. There were also two cases where the same author published two related papers. In order to reduce errors and avoid too much influence from a single author, only one of them was randomly selected for analysis in this study. In the end, we only screened 12 relevant articles for the meta-analysis. Therefore, the original data of this study came from the following 12 articles. We made Table 2 to show the relevant information of the original information sources and the data used in this study, including research objects, number of samples, acting reagents, experimental time, and research indicators. Among the articles, five articles were related to the immune index of TNF-α [26,27,28,29,30]; four articles were related to the immune index of thymus and spleen [27,31,32,33]; four articles contained the immune data of IL-10 [28,29,34,35]; and three papers contained IFN-γ [27,29,30]. IgA, IgG immune factors in serum [36,37], IL-2 [26,30], and IL-4 [29,35] in immune cells were only studied in two articles. 

### 3.2. Quality Assessment of Included Studies

After we ran statistics, one article received one point, two articles received three points, five articles received four points, and four articles received five points. Therefore, there were three low-quality documents, nine medium-quality documents, and zero high-quality documents (Table 3).

### 3.3. Meta-Analysis of Immune Indicators

**Figure 2:**
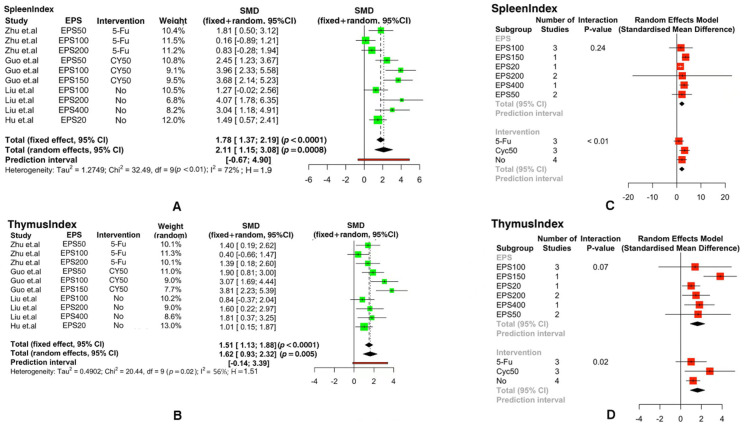
The forest plot illustrates the effects of extra-cellular polysaccharides on the immunity index of animals: (**A**) spleen index, (**B**) thymus index. Based on the above figure, a forest plot of the effect of using interfering media before EPS treatment and the effect of different concentrations of EPSs on immunity index was made: (**C**) spleen index, (**D**) thymus index. (Positive and negative values in-dicate increases or decreases in the production of immune factors. SMD: standardized mean difference.)

Indicators such as spleen index, thymus index, TNF-α, IFN-γ, IL-2, IL-4, IL-10, IgA, and IgG play an important role in the immune response to EPSs. In this work, we studied the effects of exopolysaccharides (EPSs), exopolysaccharide dosages, and intervention conditions on these immune modulators through a separate meta-analysis. The heterogeneous effects of EPSs on spleen index and thymus index were examined using either a fixed-effect model or random-effects model in the meta-analysis. Because the heterogeneities for spleen index and thymus index were I^2^ = 72%, *p* < 0.01 and I^2^ = 56%, *p* = 0.02 < 0.05, respectively, a random-effects model was used. Compared with placebo or blank reagents, oral exopolysaccharides (EPSs) significantly increased spleen index (SMD: 2.11, ‘95%CI: [1.15, 3.08]’; *p* < 0.01) and thymus index (SMD: 1.62, ‘95%CI: [0.93, 2.32]’; *p* = 0.01 < 0.05) regulation (Figure 2A,B). To further investigate the effects of different concentrations of EPSs and two types of interventions (CY and 5-Tu) on spleen index, we conducted sub-group analyses on EPS concentrations and other biological interventions. High EPS concentrations slightly increased the value of the body’s spleen index. The treatment that combined CY with EPSs had a significant effect on the increase in spleen index, while the combination of 5-Fu and EPSs rarely impacted spleen index. The effect of a single EPS treatment on spleen index was less effective than the combined CY and EPS treatment but more effective than combining 5-Fu and EPSs (Figure 2C). There was a 95% chance that the standard mean difference between EPS treatments and control groups (placebo or blank reagents) in relation to thymus index were in the interval [0.93, 2.32] (*p* < 0.05) (Figure 2B), indicating that EPSs had a significant effect on thymus index (Figure 2D). However, thymus index did not follow the increasing trend when the concentration of EPSs increased. Compared with other concentrations of EPSs, 150 mg/kg of EPSs most significantly impacted the thymus index. Interventions with CY, with 5-Fu, or without any intervention affected immunization outcomes, and the effect of CY was optimal, and the effect of 5-Fu was the lowest and was lower than that of the non-intervention group in Figure 2D.

**Figure 3:**
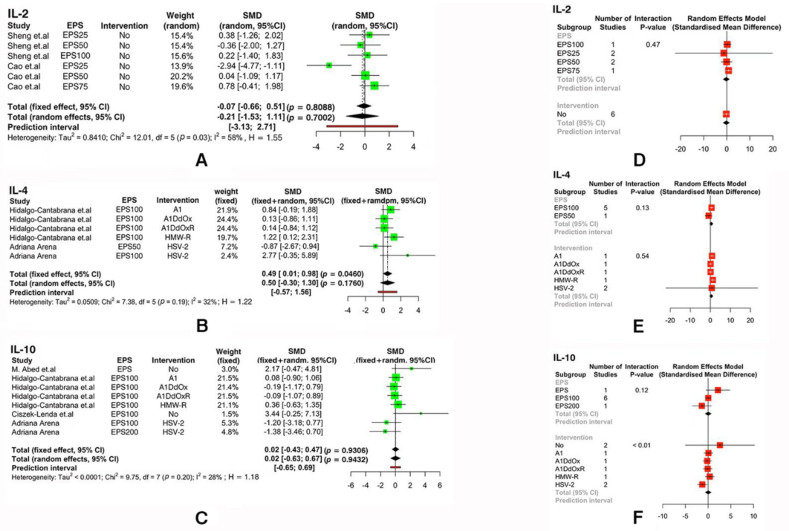
The forest plot illustrates the effects of extra-cellular polysaccharides on the immunity index of animals: (**A**) IL-2; (**B**) IL-4; (**C**) IL-10. Based on the above figure, a forest plot of the effect of using interfering media before EPS treatment and the effect of different concentrations of EPSs on immunity index was made: (**D**) IL-2; (**E**) IL-4; (**F**) IL-10. (Positive and negative values indicate increases or decreases in the production of immune factors. SMD: standardized mean difference.)

As shown in Figure 3B, due to the un-substantial heterogeneity of IL-4 (I^2^ = 32%, *p* = 0.19 > 0.05) that we observed, a fixed-effect model (SMD: 0.49, ‘95%CI: [0.01, 0.98]’; *p* = 0.046 < 0.05) was used, and it suggested that extra-cellular polysaccharides had a statistically significant effect on IL-4. However, intervention agents and the dose sub-group of extra-cellular polysaccharides had no effect on immunity (Figure 3E).

**Figure 4:**
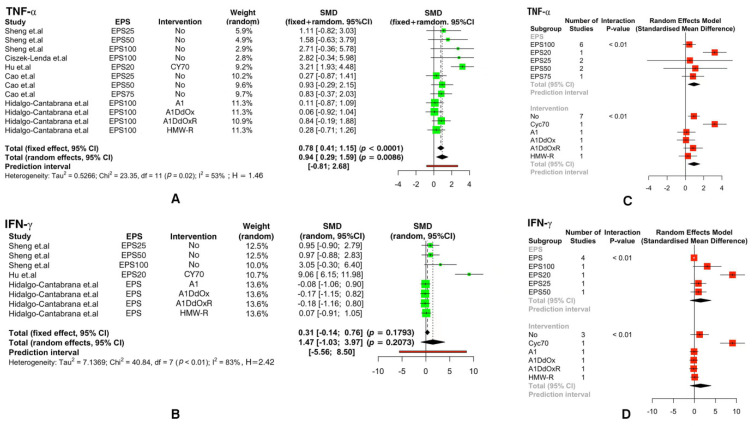
The forest plot illustrates the effects of extra-cellular polysaccharides on the immunity index of animals: (**A**) TNF-α (**B**) IFN-γ. Based on the above figure, a forest plot of the effect of using in-ter-fering media before EPS treatment and the effect of different concentrations of EPSs on immunity index was made: (**C**) TNF-α, (**D**) IFN-γ. (Positive and negative values indicate increases or de-creases in the production of immune factors. SMD: standardized mean difference).

The results related to IFN-γ (SMD: 1.47, ‘95%CI: [−1.03, 3.97]’; *p* =0.21 > 0.05) showed that extra-cellular polysaccharides had no effect on the production of IFN-γ (Figure 4B). However, EPS concentrations of 20 mg/kg were significant, and the results were significantly affected by the use of CY mixed with EPSs (Figure 4D).

EPSs had an effect on the production of TNF-α (SMD: 0.94, ‘95%CI: [0.29, 1.59]’; *p* = 0.01 < 0.05) (Figure 4A). In the sub-group studied using EPSs at different concentrations, the 95% confidence interval (CI) corresponding to the extra-cellular polysaccharide group of different concentrations intersected with 0, but the extra-cellular polysaccharide effect was most significant in the 20 mg/kg group (Figure 4C). The use of EPSs after intervention with CY (concentration of 70 mg/kg) to treat experimental animals had an impact on the experimental results, and the remaining interference conditions had no significant effect (Figure 4C).

**Figure 5:**
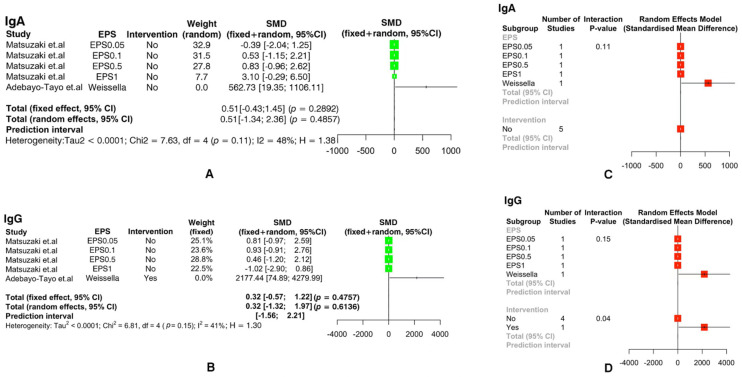
The forest plot illustrates the effects of extra-cellular polysaccharides on the immunity index of animals: (**A**) IgA, (**B**) IgG. Based on the above figure, a forest plot of the effect of using interfering media before EPS treatment and the effect of different concentrations of EPSs on immunity index was made: (**C**) IgA, (**D**) IgG. (Positive and negative values indicate increases or decreases in the production of immune factors. SMD: standardized mean difference.)

From Figure 3, Figure 4 and Figure 5, we found that extracellular polysaccharides had no effect on IL-2 (SMD: -0.21, ‘95%CI: [−1.53, 1.11]’; *p* = 0.70 > 0.05), IL-10 (SMD: 0.02, ‘95%CI: [−0.43, 0.47]’; *p* = 0.93 > 0.05), IgA (SMD: 0.51, ‘95%CI: [−0.43, 1.45]’; *p* = 0.29 > 0.05), and IgG (SMD: 0.32, ‘95%CI: [−0.57, 1.22]’; *p* = 0.48 > 0.05), and sub-groups of EPSs with different concentrations and sub-groups with different intervention conditions also had no effect on the experimental group.

## 4. Discussion

This meta-analysis investigated the effects of different sources of EPSs on immune regulation, and it involved a total of nine relevant immune indices. Knowing that different structures and different sources of EPSs have great differences in the regulation of immunity, we evaluated the effects of different doses and types of EPSs on inflammatory mediators. We conducted research on sub-groups and found that EPSs had a significant effect on the content of experimental spleen and thymus immune index. However, according to our analysis of Figure 2C,D, we found that different concentrations of EPSs had a significant impact on experiments, but the actual datasheet did not follow the expected results of spleen index and thymus index increasing accordingly as the EPS concentration increased. As shown in Figure 2C,D, the increase in exopolysaccharide-regulated thymic immune index showed an irregular trend. It may be that EPSs have a suitable concentration range requirement to achieve the best effect on spleen index. Alternatively, we may have had a heterogeneous dataset. However, when each study was analyzed, the immune index for some experiments generally increased with increases in EPS concentration. The above reasons for the irregular change in the immune index effect value with the increase in concentration may be because the purity of extracted EPSs is different in different experiments. Secondly, the types of EPSs extracted were also extremely different, resulting in a large difference in immune stimulation using the same concentration of EPSs.

The reason for the difference between different types of EPSs may be that the components of EPSs, glycoside bonds, etc., are different, and the recognition effect of the surface receptors of immune cells is different [38]. β-1,3 and β-1,6 glycosidic linked glucans are recognized by Dectin receptors to promote the release of inflammatory mediators in macrophages [39]. They contain mannose residues, trehalose groups, etc., that can be recognized by mannan receptors, an important member of the C-type lectin-like receptor family, and they act on macrophages, play an important role in early immune responses, and activate cytokines [40].

In the sub-group analysis, the treatment of study subjects with different intervention agents had an impact on the immunomodulatory effect of extra-cellular polysaccharides. In Figure 2C,D, CY was used to intervene in experiments, and the effect of extra-cellular polysaccharides was relatively significant, and 5-Fu was the least significant. In Figure 2C,D, the effect of extra-cellular polysaccharides was relatively significant when CY was used to intervene in experiments compared to the group without intervention agents. However, with 5-Fu, the effect of extra-cellular polysaccharides was lower than that of the non-intervention group. The reason for the above phenomenon may be that after the intervention agent stimulated the study subject, the body’s immune level was different, resulting in heterogeneity in the experience results. It may be because 5-Fu has a higher ability to suppress animal immunity, so the body’s immunity level is low after 5-Fu intervention (even lower than that of a non-intervention group). After EPS action, the immune level did not change much, and the effect was not obvious compared with other intervention groups. However, due to the lack of a large number of experimental samples with the same interference conditions, this conclusion needs more experimental samples to be verified.

EPSs also had a significant effect on the increase in thymus immune index. The correlation of thymus index was roughly the same as that of spleen index, but the effect of EPSs on the increase in thymus index was better than that of spleen. A large number of studies have also shown that EPSs have good immune regulation function, which can activate natural T cells, induce the proliferation of T cells, and produce cytokines, which may be the reason why the immune index of the thymus was higher than that of the spleen. Epimedium polysaccharide improves T cell immune mediation by increasing the number of CD3^+^ and CD4^+^ cells and the ratio of CD4^+^/CD8^+^, thereby enhancing immune function and reducing hematologic toxicity in mice with benzene-induced bone marrow failure [41]. When the EPSs produced by *Lactobacillus casei* are recognized by antigen-presenting cells (APCs), they induce the differentiation of CD4^+^ T cells into Th17 cells, and the T cell effectors secreted by *Lactobacillus casei* can clear the antigen and play a role in the host immune response. At the same time, DC cells gradually mature after ingesting antigens and secrete cytokines, which can also stimulate the activation of natural T cells [42].

From the meta-analysis forest plot, we observed that EPS had no effect on IFN-γ (Figure 4), and neither did other immune factor indicators (IgA, IgG, IL-2, IL-4, and IL-10) in this meta-analysis (Figure 3 and Figure 5). EPSs were able to promote IFN-γ production in mice in every experiment. However, experimental errors may have result in large differences among the included data, which leads to insignificant results. The effect of 20 mg/kg of EPS on IFN-γ and TNF-α was the most prominent, but we cannot rule out the error caused by the above reasons. After examination, the data using 20 mg/kg of EPSs all came from the same articles, which once again verified the reasons for the heterogeneity in the above experimental results.

For heterogeneity reasons, the results for IL-2, IL-4, IL-10, IFN-γ, IgA, and IgG were not ideal. Below, we cite some EPS studies on immunomodulation to demonstrate the regulatory power of EPSs with respect to these immune factors. Many experiments were conducted to study the function of EPSs. Xiu et al. [43] found that probiotic *Lactobacillus casei* WXD030 EPSs enhanced the content of IFN-γ in CD4^+^ T cells. Oral administration of EPSs extracted from yogurt fermented with *Lactobacillus delbrueckii* ssp. enhances natural killer cell activity and induces IFN-γ production by splenocytes [44]. According to Kong et al. [45], black fungus polysaccharide has an immunomodulatory effect on mice treated with cyclophosphamide, and the levels of IFN-γ, IL-2, IL-4, and IL-10 in the serum of mice are significantly up-regulated. These EPSs all promote the production of IFN-γ for immune regulation, which is consistent with the results analyzed in this paper. EPSs isolated from hydrothermal strains induce macrophages to produce the pro-inflammatory cytokines TNF-α and IL-6 by stimulating Toll-Like Receptor 2 (TLR2 receptors) [46]. Matsuzaki et al. [47] demonstrated that EPSs extracted from *Leuconostoc mesenteroides* strain NTM048 were able to induce lgA and IgG production. *Aspergillus terreus* EPSs can activate macrophages and promote the production of NO, TNF-α, and IL-6 [48]. Black fungus polysaccharide also has immunomodulatory function, which can promote the release of NO, TNF-α, IL-6, IL-10, and other cytokines from macrophages [49]. As reported by kuang et al. [50], *Bacillus amyloliquefaciens* DMBA-K4 EPSs can alleviate the symptoms of colitis and promote the expression of the anti-inflammatory cytokine IL-10 in mice. These studies have demonstrated, to some extent, that EPSs can promote IL-2, IL-4, IL-10, IFN-γ, IgA, and IgG to regulate immunity, which is consistent with our conjectures about the immunomodulatory efficacy of EPSs.

IgA and IgG immunoglobulin are major players in the adaptive immune response, which contributes to immune defense and maintains homeostasis, and the binding of IgA to the Fc receptor (FcαRI) can trigger a pro-inflammatory or anti-inflammatory response [51]. TNF-α, IFN-γ, and IL-2 belong to pro-inflammatory factors. Like TNF-α, they are key components of normal immunity and can activate immune regulation. However, inappropriate or excessive production of TNF-α may also be harmful and may cause chronic inflammation [52]. IL-4 and IL-10 are anti-inflammatory factors. For example, IL-4 plays a key role in Th2 differentiation. It can resist the pro-inflammatory immune response driven by Th1. Moreover, it can down-regulate the synthesis of many pro-inflammatory factors and inhibit pro-inflammatory chemokines and other inflammatory mediators [53].These inflammatory factors are closely related to the inflammation of the body. Inflammation is the response of the immune system to harmful stimuli, such as pathogens, damaged cells, toxic compounds, or irradiation, and it acts by removing harmful stimuli and initiating the healing process [54].

Inflammation is a vital defense mechanism for health. During acute inflammatory responses, cell and molecular interactions minimize impending injury or infection [55]. These actions include changes in vascular permeability, leukocyte recruitment and accumulation, and mediator release during inflammation [56]. Inflammation stimulates intra-cellular signaling pathways, including the mitogen-activated protein kinase (MAPK) pathway, the nuclear factor kappa-B (NF-κB) pathway, and the Janus kinase (JAK)-signal transducer and activator of transcription (STAT) pathways, and then, it activates the production of inflammatory mediators [57,58,59]. Like IFN- γ, for TNF- α, IL-1 β, IL-6, IL-8, IL-12, and other pro-inflammatory factors, the core of their production is the activation of sensors, such as toll like receptors (TLRs), and they activate inflammatory stimulation signal channels to promote the production of cytokines and the recruitment of inflammatory cells, which contribute to the inflammatory response [60]. Although inflammation responds to infection and tissue damage, which helps to restore tissue homeostasis and the regression of acute inflammation, the excessive production of inflammatory mediators can lead to harmful results from inflammatory diseases [61]. Uncontrolled acute inflammation may become chronic inflammation, leading to a variety of chronic inflammatory diseases [55]. Cardiovascular disease and arteriosclerosis are the main causes of death and disability in the world. From the initial leukocyte recruitment to the rupture of atherosclerotic plaque, inflammatory mediators play a key role in arteriosclerosis [62,63]. However, the anti-inflammatory factors IL-10 can inhibit inflammation, enhance the regulation of the myofibroblast phenotype, and promote the deposition of extra-cellular matrix to promote cardiac repair [64]. In the regression stage of inflammation, macrophages produce anti-inflammatory mediators, such as IL-10 and TGF-β, and a variety of growth factors, such as insulin-like growth factor -1 (IGF-1) and vascular endothelial growth factor-α (VEGF-α). These can promote fibroblast differentiation, reshape extracellular matrix components, and stimulate collagen synthesis, angiogenesis, etc., and they promote the complete restoration of tissue structure and function [64,65]. 

Inhibiting inflammation means reducing the unnecessary side effects caused by the excessive production of inflammatory mediators; regulating the balance between pro-inflammatory and anti-inflammatory factors; and achieving the purpose of promoting tissue healing, repairing function, and regulating adaptive immunity. In regulating immune balance, targeting inflammatory factors can not only promote the production of anti-inflammatory factors, but it can also reduce the number of relevant pro-inflammatory factors. Hoang et al. [66] found that seaweed polysaccharide inhibits the synthesis of iNOS, TNF-α, IL-6, and IL-8. The acidic exopolysaccharide of marine amyloid *Bacillus* can inhibit cyclooxygenas (COX)-2 enzymes involved in inflammation [67]. In this meta-analysis, under the fixed-effect model, the synthesis of the anti-inflammatory factor IL-4 increased after the action of EPSs, which is of great significance. IL-10, also as an anti-inflammatory cytokine, may also be moderated in expression. This result is consistent with the changes in inflammatory factors in mice when inflammation subsides. EPSs can regulate the synthesis of mouse cell pro-inflammatory factors and activate relevant pathways to remove harmful stimuli. They can also promote the synthesis of anti-inflammatory factors when inflammation subsides, so as to adjust the body’s immunity and achieve immune balance. It can be seen that extra-cellular polysaccharides have good immune regulation ability.

In this study, the included studies had significant heterogeneity in methodology and statistics. There was obvious methodological heterogeneity among different types of biological enhancement techniques and types, purity, samples of tested animals, inflammatory status, and research time using extra-cellular polysaccharides. Second, a large number of articles in this review were of medium quality, and most experiments were conducted on mice; due to the lack of experimental data, there was an article on human cells was included in the study, which could have increased heterogeneity. These inevitable factors did cause a non-negligible interference in our meta-analysis. However, when we collect data, we try to limit heterogeneity caused by some non-essential factors through strict inclusion and exclusion criteria. For example, in these experiments, EPSs were used alone to treat subjects without the influence of other drug factors. In this meta-analysis, we performed repeated screening, study selection, data extraction, and rigorous quality assessments to minimize errors to improve accuracy. We used the forest plots to analyze the significance of each sub-group. For the topics lacking in research data, we analyzed the data under the appropriate model to reduce deviations and get closer to achieving accurate analysis results. Moreover, this study used a large number of reports on EPS function to further support our conclusions and make the analysis more reliable. Finally, the analysis confirmed that EPSs have a significant effect on immunomodulation.

## 5. Conclusions

In summary, this meta-analysis confirmed that EPSs, whether from bacteria or fungi, have good immunomodulatory effects. However, given the heterogeneity present in our meta-analysis, additional evidence from a large number of well-designed, long-term experiments is still needed for further validation. This study confirmed the good immunomodulatory effects of EPSs, making them more applicable. Further investigations on EPSs are needed to examine the potential for widely using EPSs in food and pharmaceutical industries. Interestingly, from our study, we found that the effect of EPSs with different concentrations on immune modulators were varied to some extent. Therefore, it is important to further study the effects of different types of EPSs on immune function, especially at the molecular level. In the future, for one example, we should work on engineering more robust glycoside bonds and groups in EPS structure to develop extra-cellular polysaccharides with better immune effects for food applications.

## Figures and Tables

**Table 1:** PICOS criteria for inclusion of studies.

Parameter	Study
Population	Mice, people
Intervention	EPSs from Fungi, bacteria, mold; any time, dosage, or form
Comparator	Placebo (with any co-intervention)
Outcomes	IFN-γ, TNF-α, IL-2, IL-4, IL-10, IgG, IgA, Thymus index, Splenomeric index
Study	Randomized controlled trials, published in English

**Table 2:** Main characteristics of eligible trials.

Data from	Research Objects	Number	Treatment Duration	Medicament	Research Indicators
Reprinted/adapted with permission from Ref. [26]. 2016, Jianfeng Cao	Mouse serum, spleen, and thymus from S180 tumor-bearing mice	① *n* = 7	① 17 d	①, ② 25, 50, 75 mg/kg EPS from *Rhizopus nigricans* Ehrenb; S180 tumor-bearing mice	Spleen and thymus index, IL-2, TNF-a
		② *n* = 6	② 10 d		
Reprinted/adapted with permission from Ref. [27]. 2016, Ting Hu	Mouse serum, Spleen, and thymus of mice	*n* = 12	8 d	EPSs of a Cordyceps sinensis fungus (20 mg/kg); cyclophosphamide-induced-mice (70 mg/kg)	Spleen and thymus index, IL-10, TNF-α, INF-γ
Reprinted/adapted with permission from Ref. [28]. 2011, Marta Ciszek-Lenda	Mouse macrophage	5 × 10^5^ cell/mL	1 d	LPS (1 μg/mL); *Lactobacillus rhamnosus* KL37, *Escherichia coli* 0111:B4, whole heat-killed bacteria, EPSs from *Lactobacillus rhamnosus* KL37(100 μg/mL)	TNF-α, IL-6, IL-10, IL-12p40
Reprinted/adapted with permission from Ref. [29]. 2014, Claudio Hidalgo-Cantabrana	Immune cells isolated from mouse GALT and PBMC	*n* = 8 2 × 10^6^ cell/mL	5.25 d	EPSs of sub-species Bifidobacterium (100 μg/mL) PHA (2.5 μg/mL)	IFNγ, IL-10, IL-1a, IL-4, TNF-α, IL-17, TGF-β
Reprinted/adapted with permission from Ref. [30]. 2011, Lu Sheng	Splenocytes, thymocytes	①, ②: *n* = 6 1 × 10^6^ cell/mL	① 1 d, 2 d ② 20 h	① Add 10 μg of the mitogen concanavalin A and different doses of EPSs (the ultimate concentrations were 12.5, 25, 50, 100 μg/mL, respectively) ② Treat with 25, 50, 100 μg/mL of Cordyceps sinensis	Splenocytes, thymocytes, TNF-α, IFN-γ, IL-2
Reprinted/adapted with permission from Ref. [31]. 2010, Jun Liu	Spleen and thymus of mice	*n* = 6	42 d	EPS from endophytic bacterium *Paenibacillus polymyxa* EJS-3 (100, 200, 400 mg/kg body weight per day); d-Gal-induced mice (100 mg/kg)	Spleen and thymus index
Reprinted/adapted with permission from Ref. [32]. 2013, Yuxing Guo	Spleen and thymus of mice	*n* = 10	2 d	Selenium EPS produced by *Lactococcus lactis* subsp. *Lactis* (50, 100, 150 mg/kg); EPSs produced by *Lactococcus lactis* subsp. *Lactis* (50, 100, 150 mg/kg); CY treatment of mice (50 mg/kg)	Spleen and thymus index
Reprinted/adapted with permission from Ref. [33]. 2016, Lei Zhu	Thymus and spleen of mice	*n* = 7	14 d	5-Fu, (20 mg/kg); EPS from *Rhizopus nigricans*, at the doses of 50, 100, 200 mg/kg combined with 5-Fu at a dose of 20 mg/kg	Index and weight for thymus and spleen
Reprinted/adapted with permission from Ref. [34]. 2020, Saif M. Abed	Mouse serum	Triple dilution	14 d	EPSs produced from Vancomycin Resistant Enterococcus faecalis (0.5, 1 mg/mL)	IL-10
Reprinted/adapted with permission from Ref. [35]. 2009, Adriana Arena	Human PMBC	2 × 10^6^ cell/mL	1 d	PBMC was added with EPSs (0, 50, 100, 200, and 300 μg/mL);	IFN-α, TNF-α, IL-4, IL-10, IL-12, IL-18
Reprinted/adapted with permission from Ref. [36]. 2015, Chiaki Matsuzaki	Rat plasma	*n* = 4	42 d	Add ad labium the NTM048 EPS-containing experimental water at a concentration of 0%, 0.05%, 0.1%, 0.5%, or 1% *w*/*v*	IgA, IgG
Reprinted/adapted with permission from Ref. [37]. 2020, Bukola Adebayo-Tayo	Mouse serum	*n* = 4 Diluted 10 times normal saline	15 d	Cigarettes; 20 mg/kg/day 5-fluorouracil; with 500 nM aqueous solution of EPSWLD or EPSMLD through intra-peritoneal (IP) administration	IgG, IgA, IgM

**Table 3:** Quality assessment analysis form.

Study	①	②	③	④	⑤	⑥	⑦	⑧	⑨	Score
Reprinted/adapted with permission from Ref. [26]. 2016, Jianfeng Cao	✓	-	✓	-	-	-	-	✓	✓	4
Reprinted/adapted with permission from Ref. [27]. 2016, Ting Hu	✓	-	✓	-	-	✓	-	✓	✓	5
Reprinted/adapted with permission from Ref. [28]. 2011, Marta Ciszek-Lenda	✓	-	-	-	-	-	-	✓	✓	3
Reprinted/adapted with permission from Ref. [29]. 2014, Claudio Hidalgo-Cantabrana	✓	-	-	-	-	✓	-	✓	✓	4
Reprinted/adapted with permission from Ref. [30]. 2011, Lu Sheng	✓	-	-	-	-	-	-	✓	✓	3
Reprinted/adapted with permission from Ref. [31]. 2010, Jun Liu	✓	-	✓	-	-	✓	-	✓	✓	5
Reprinted/adapted with permission from Ref. [32]. 2013, Yuxing Guo	✓	-	✓	-	-	✓	-	✓	✓	5
Reprinted/adapted with permission from Ref. [33]. 2016, Lei Zhu	✓	-	-	-	-	✓	-	✓	✓	4
Reprinted/adapted with permission from Ref. [34]. 2020, Saif M. Abed	✓	-	✓	-	-	✓	-	✓	✓	5
Reprint-ed/adapted with permission from Ref. [35]. 2009, Adriana Arena	✓					✓		✓	✓	4
Reprinted/adapted with permission from Ref. [36]. 2015, Chiaki Matsuzaki	✓	-	-	-	-	✓	-	✓	✓	4
Reprinted/adapted with permission from Ref. [37]. 2020, Bukola Adebayo-Tayo	-	-	-	-	-	-	-	-	✓	1

## Data Availability

The authors confirm that the data supporting the results of this study are available in articles, reference publications. The data for this article is available on the publisher’s website.

## References

[1] Zannini E., Waters D.M., Coffey A., Arendt E.K. (2016). Production, properties, and industrial food application of lactic acid bacteria-derived exopolysaccharides. Appl. Microbiol. Biot..

[2] London L., Chaurin V., Auty M., Fenelon M.A., Fitzgerald G.F., Ross R.P., Stanton C. (2015). Use of *Lactobacillus mucosae* DPC 6426, an exopolysaccharide-producing strain, positively influences the techno-functional properties of yoghurt. Int. Dairy J..

[3] Franck A. (2002). Technological functionality of inulin and oligofructose. Br. J. Nutr..

[4] Carboni E., Tschudi K., Nam J., Lu X., Ma A.W. (2014). Particle margination and its implications on intravenous anticancer drug delivery. AAPS PharmSciTech.

[5] Kawashima T., Murakami K., Nishimura I., Nakano T., Obata A. (2012). A sulfated polysaccharide, fucoidan, enhances the immunomodulatory effects of lactic acid bacteria. Int. J. Mol. Med..

[6] Gugliandolo C., Spanò A., Maugeri T.L., Poli A., Arena A., Nicolaus B. (2015). Role of bacterial exopolysaccharides as agents in counteracting immune disorders induced by herpes virus. Microorganisms.

[7] Reisacher W.R., Davison W.N.S. (2017). Immunotherapy for food allergy. Curr. Opin. Otolaryngol. Head Neck. Surg..

[8] Luo M., Gan M., Yu X., Wu X., Xu F. (2020). Study on the regulatory effects and mechanisms of action of bifidobacterial exopolysaccharides on anaphylaxes in mice. Int. J. Biol. Macromol..

[9] Górska S., Schwarzer M., Srutkova D., Hermanova P., Brzozowska E., Kozakova H., Gamian A. (2017). Polysaccharides L900/2 and L900/3 isolated from *Lactobacillus rhamnosus* LOCK 0900 modulate allergic sensitization to ovalbumin in a mouse model. Microb. Biotechnol..

[10] Bisht V., Lal B. (2019). Exploration of performance kinetics and mechanism of action of a potential novel bioflocculant BF-VB2 on clay and dye wastewater flocculation. Front. Microbiol..

[11] Park Y., Hong Y.N., Weyers A., Kim Y.S., Linhardt R.J. (2011). Polysaccharides and phytochemicals: A natural reservoir for the green synthesis of gold and silver nanoparticles. IET Nanobiotechnol..

[12] Tahmourespour A., Ahmadi A., Fesharaki M. (2020). The anti-tumor activity of exopolysaccharides from *Pseudomonas* strains against HT-29 colorectal cancer cell line. Int. J. Biol. Macromol..

[13] Ismail B., Nampoothiri K. (2013). Exposition of antitumour activity of a chemically characterized exopolysaccharide from a probiotic *Lactobacillus plantarum* MTCC 9510. Biologia.

[14] Yilmaz T., Şimşek Ö. (2020). Potential health benefits of ropy exopolysaccharides produced by *Lactobacillus plantarum*. Molecules.

[15] Zhou Y., Cui Y., Suo C., Wang Q., Qu X. (2021). Structure, physicochemical characterization, and antioxidant activity of the highly arabinose-branched exopolysaccharide EPS-M2 from *Streptococcus thermophilus* CS6. Int. J. Biol. Macromol..

[16] Sun H., Yu X., Li T., Zhu Z. (2021). Structure and hypoglycemic activity of a novel exopolysaccharide of *Cordyceps militaris*. Int. J. Biol. Macromol..

[17] Gong G., Dang T., Deng Y., Han J., Zou Z., Jing S., Zhang Y., Liu Q., Huang L., Wang Z. (2018). Physicochemical properties and biological activities of polysaccharides from *Lycium barbarum* prepared by fractional precipitation. Int. J. Biol. Macromol..

[18] Ferreira S.S., Passos C.P., Madureira P., Vilanova M., Coimbra M.A. (2015). Structure-function relationships of immunostimulatory polysaccharides: A review. Carbohydr. Polym..

[19] Di W., Zhang L.W., Wang S.M., Yi H.X., Han X., Fan R.B., Zhang Y.C. (2017). Physicochemical characterization and antitumour activity of exopolysaccharides produced by *Lactobacillus casei* SB27 from yak milk. Carbohydr. Polym..

[20] Surayot U., Wang J., Seesuriyachan P., Kuntiya A., Tabarsa M., Lee Y., Kim J.K., Park W., You S. (2014). Exopolysaccharides from lactic acid bacteria: Structural analysis, molecular weight effect on immunomodulation. Int. J. Biol. Macromol..

[21] Xiao L., Han S., Zhou J., Xu Q., Dong M., Fan X., Rui X., Chen X., Zhang Q., Li W. (2020). Preparation, characterization and antioxidant activities of derivatives of exopolysaccharide from *Lactobacillus helveticus* MB2-1. Int. J. Biol. Macromol..

[22] Moher D., Liberati A., Tetzlaff J., Altman D.G. (2009). Preferred reporting items for systematic reviews and meta-analyses: The PRISMA statement. Ann. Intern Med..

[23] Hedges L., Olkin I. (1985). Statistical Methods in Meta-Analysis.

[24] Gregory P.T. (2004). Analysis of patterns of aggregation under cover objects in an assemblage of six species of snakes. Herpetologica.

[25] Higgins J.P.T., Thompson S.G. (2002). Quantifying heterogeneity in a meta-analysis. Stat. Med..

[26] Cao J., Hou D., Lu J., Zhu L., Zhang P., Zhou N., Chen K. (2016). Anti-tumor activity of exopolysaccharide from *Rhizopus nigricans* Ehrenb on S180 tumor-bearing mice. Bioorg. Med. Chem. Lett..

[27] Hu T., Jiang C., Huang Q., Sun F. (2016). A comb-like branched β-D-glucan produced by a *Cordyceps sinensis* fungus and its protective effect against cyclophosphamide-induced immunosuppression in mice. Carbohydr. Polym..

[28] Ciszek-Lenda M., Nowak B., Srottek M., Gamian A., Marcinkiewicz J. (2011). Immunoregulatory potential of exopolysaccharide from *Lactobacillus rhamnosus* KL37. effects on the production of inflammatory mediators by mouse macrophages. Int. J. Exp. Pathol..

[29] Hidalgo-Cantabrana C., Nikolic M., López P., Suárez A., Miljkovic M., Kojic M., Margolles A., Golic N., Ruas-Madiedo P. (2014). Exopolysaccharide-producing *Bifidobacterium animalis* subsp. lactis strains and their polymers elicit different responses on immune cells from blood and gut associated lymphoid tissue. Anaerobe.

[30] Sheng L., Chen J., Li J., Zhang W. (2011). An exopolysaccharide from cultivated *Cordyceps sinensis* and its effects on cytokine expressions of immunocytes. Appl. Biochem. Biotechnol..

[31] Liu J., Luo J.G., Ye H., Sun Y., Lu Z.X., Zeng X.X. (2010). *In vitro* and *in vivo* antioxidant activity of exopolysaccharides from endophytic bacterium *Paenibacillus polymyxa* EJS-3. Carbohydr. Polym..

[32] Guo Y., Pan D., Li H., Sun Y., Zeng X. (2013). Antioxidant and immunomodulatory activity of selenium exopolysaccharide produced by *Lactococcus lactis* subsp. lacti. Food Chem..

[33] Zhu L., Cao J.F., Chen G.C., Xu Y.H., Lu J.B., Fang F., Chen K.S. (2016). Anti-tumor and immunomodulatory activities of an exopolysaccharide from *Rhizopus nigricans* on CT26 tumor-bearing mice. Int. Immunopharmacol..

[34] Abed S., Essa R.H., Alaraji K.H.Y. (2020). Evaluation of the antibacterial activity and immunomodulatory effect of purified exopolysaccharides (EPSs) produced from vancomycin resistant *Enterococcus faecalis*. Int. J. Drug Deliv. Tec..

[35] Arena A., Gugliandolo C., Stassi G., Pavone B., Iannello D., Bisignano G., Maugeri T.L. (2009). An exopolysaccharide produced by *Geobacillus thermodenitrificans* strain B3-72: Antiviral activity on immunocompetent cells. Immunol. Lett..

[36] Matsuzaki C., Hayakawa A., Matsumoto K., Katoh T., Yamamoto K., Hisa K. (2015). Exopolysaccharides produced by *Leuconostoc mesenteroides* strain NTM048 as an immunostimulant to enhance the mucosal barrier and influence the systemic immune response. J. Agric. Food. Chem..

[37] Adebayo-Tayo B., Fashogbon R. (2020). *In vitro* antioxidant, antibacterial, *in vivo* immunomodulatory, antitumor and hematological potential of exopolysaccharide produced by wild type and mutant *Lactobacillus delbureckii* subsp. bulgaricus. Heliyon.

[38] Wang M., Yang X.B., Zhao J.W., Lu C.J., Zhu W. (2017). Structural characterization and macrophage immunomodulatory activity of a novel polysaccharide from *Smilax glabra Roxb*. Carbohydr. Polym..

[39] Lee J.-B., Tanikawa T., Hayashi K., Asagi M., Kasahara Y., Hayashi T. (2015). Characterization and biological effects of two polysaccharides isolated from *Acanthopanax sciadophylloides*. Carbohydr. Polym..

[40] Li W.J., Tang X.F., Shuai X.X., Jiang C.J., Xie M.Y. (2017). Mannose receptor mediates the immune response to *Ganoderma atrum* polysaccharides in macrophages. J. Agric. Food Chem..

[41] He J., Zang S.L., Liu N., Ji M., Ma D.X., Ji C.Y. (2020). Epimedium polysaccharides attenuates hematotoxicity by reducing oxidative stress and enhancing immune function in mice model of benzene-induced bone marrow failure. Biomed. Pharmacother..

[42] Ren Q., Tang Y., Zhang L., Xu Y., Liu N., Ren H. (2020). Exopolysaccharide produced by *Lactobacillus casei* promotes the differentiation of CD4(+) T cells into Th17 Cells in BALB/c mouse peyer’s patches *in vivo* and *in vitro*. J. Agric. Food Chem..

[43] Xiu L., Zhang H.C., Hu Z.P., Liang Y.C., Guo S., Yang M., Du R.P., Wang X. (2018). Immunostimulatory activity of exopolysaccharides from probiotic *Lactobacillus casei* WXD030 strain as a novel adjuvant *in vitro* and *in vivo*. Food Agric. Immunol..

[44] Makino S., Sato A., Goto A., Nakamura M., Ogawa M., Chiba Y., Hemmi J., Kano H., Takeda K., Okumura K. (2016). Enhanced natural killer cell activation by exopolysaccharides derived from yogurt fermented with *Lactobacillus delbrueckii* ssp. bulgaricus OLL1073R-1. J. Dairy Sci..

[45] Kong X.H., Duan W.W., Li D.J., Tang X.X., Duan Z.H. (2020). Effects of polysaccharides from *Auricularia auricula* on the immuno-stimulatory activity and gut microbiota in immunosuppressed mice induced by cyclophosphamide. Front. Immunol..

[46] Lin M.H., Yang Y.L., Chen Y.P., Hua K.F., Lu C.P., Sheu F., Lin G.H., Tsay S.S., Liang S.M., Wu S.H. (2011). A novel exopolysaccharide from the biofilm of *Thermus aquaticus* YT-1 induces the immune response through Toll-like receptor 2. J. Biol. Chem..

[47] Matsuzaki C., Kamishima K., Matsumoto K., Koga H., Katayama T., Yamamoto K., Hisa K. (2014). Immunomodulating activity of exopolysaccharide-producing *Leuconostoc mesenteroides* strain NTM048 from green peas. J. Appl. Microbiol..

[48] Costa C.R.L.D., Menolli R.A., Osaku E.F., Tramontina R., de Melo R.H., do Amaral A.E., Duarte P.A.D., de Carvalho M.M., Smiderle F.R., Silva J.L.D. (2019). Exopolysaccharides from *Aspergillus terreus*: Production, chemical elucidation and immunoactivity. Int. J. Biol. Macromol..

[49] Zhang Y., Zeng Y., Men Y., Zhang J., Liu H., Sun Y. (2018). Structural characterization and immunomodulatory activity of exopolysaccharides from submerged culture of *Auricularia auricula-judae*. Int. J. Biol. Macromol..

[50] Kuang J.H., Huang Y.Y., Hu J.S., Yu J.J., Zhou Q.Y., Liu D.M. (2020). Exopolysaccharides from *Bacillus amyloliquefaciens* DMBA-K4 ameliorate dextran sodium sulfate-induced colitis via gut microbiota modulation. J. Funct. Foods.

[51] van Gool M.M.J., van Egmond M. (2020). IgA and FcαRI: Versatile players in homeostasis, infection, and autoimmunity. Immunotargets Ther..

[52] Bradley J.R. (2008). TNF-mediated inflammatory disease. J. Pathol..

[53] Paul W.E. (2015). History of interleukin-4. Cytokine.

[54] Ferrero-Miliani L., Nielsen O.H., Andersen P.S., Girardin S.E. (2007). Chronic inflammation: Importance of NOD2 and NALP3 in interleukin-1beta generation. Clin. Exp. Immunol..

[55] Zhou Y., Hong Y., Huang H. (2016). Triptolide Attenuates inflammatory response in membranous glomerulo-nephritis rat via downregulation of NF-κB signaling pathway. Kidney Blood Press Res..

[56] Takeuchi O., Akira S. (2010). Pattern recognition receptors and inflammation. Cell.

[57] Kaminska B. (2005). MAPK signalling pathways as molecular targets for anti-inflammatory therapy--from molecular mechanisms to therapeutic benefits. Biochim. Biophys. Acta.

[58] O’Shea J.J., Schwartz D.M., Villarino A.V., Gadina M., McInnes I.B., Laurence A. (2015). The JAK-STAT pathway: Impact on human disease and therapeutic intervention. Annu. Rev. Med..

[59] Henríquez-Olguín C., Altamirano F., Valladares D., López J., Allen P.D., Jaimovich E. (2015). Altered ROS production, NF-κB activation and Interleukin-6 gene expression induced by electrical stimulation in dystrophic mdx skeletal muscle cells. Biochim. Biophys. Acta.

[60] Hayden M.S., Ghosh S. (2012). NF-κB, the first quarter-century: Remarkable progress and outstanding questions. Genes Dev..

[61] Okin D., Medzhitov R. (2012). Evolution of inflammatory diseases. Curr. Biol..

[62] Sofi F., Fabbri A., Casini A., Romagnolo D.F., Selmin O.I. (2016). Mediterranean Diet: Dietary Guidelines and Impact on Health and Disease.

[63] Libby P., Okamoto Y., Rocha V.Z., Folco E. (2010). Inflammation in atherosclerosis: Transition from theory to practice. Circ. J..

[64] Kaur K., Dhingra S., Slezak J., Sharma A.K., Bajaj A., Singal P.K. (2009). Biology of TNFalpha and IL-10, and their imbalance in heart failure. Heart Fail Rev..

[65] Huang S., Frangogiannis N.G. (2018). Anti-inflammatory therapies in myocardial infarction: Failures, hopes and challenges. Br. J. Pharmacol..

[66] Hoang M.H., Kim J.Y., Lee J.H., You S., Lee S.J. (2015). Antioxidative, hypolipidemic, and anti-inflammatory activities of sulfated polysaccharides from *Monostroma nitidum*. Food Sci. Biotechnol..

[67] El-Newary S.A., Ibrahim A.Y., Asker M.S., Mahmoud M.G., El Awady M.E. (2017). Production, characterization and biological activities of acidic exopolysaccharide from marine *Bacillus amyloliquefaciens* 3MS 2017. Asian Pac. J. Trop. Med..

